# Gender differences in depression, anxiety, and stress during the first wave of the COVID-19 pandemic in serbia - results from an online survey

**DOI:** 10.1192/j.eurpsy.2021.726

**Published:** 2021-08-13

**Authors:** A. Opanković, M. Latas, S. Jerotić, I. Ristić, S. Milovanović

**Affiliations:** 1 Psychiatry, Clinical Centre of Serbia, Belgrade, Serbia; 2 Psychiatry, University of Belgrade, Faculty of Medicine, Belgrade, Serbia

**Keywords:** COVID-19, Gender differences, DASS-21, Anxiety

## Abstract

**Introduction:**

COVID-19 outbreak has significantly affected the mental health of people worldwide. The first wave of the pandemic began during the beginning of March 2020, and included significant preventative measures. Previous research on mental health differences between the genders has found that women were more likely to exhibit symptoms of anxiety during the COVID-19 outbreak.

**Objectives:**

The aim of our study was to assess the differences between the genders in depression, anxiety, and stress.

**Methods:**

An online survey designed for the purposes of this study was distributed using the snowballing method during April and May of 2020. The survey consisted of two parts: sociodemographic and clinical data, and COVID-19 related data. Participants also filled out the Depression, Anxiety and Stress Scale (DASS-21) - a well validated, self-questionnaire instrument. T-test for independent samples was used to evaluate the differences between the genders.

**Results:**

Out of the initially sent 563 surveys, a total of 161 were included in further analyses (28.6% response rate). Mean age of the sample was 42.2±10.2 and 65.2% of the participants were female. Mean scores on DASS-21 were as follows: 8.78±6.66 on depression, 9.78±7.39 on anxiety and 7.76±7.14 on stress. Females scored significantly higher on depression (t=-2.25, p=0.029), anxiety (t=-2.25, p=0.028), stress (t=-2.44, p=0.017), and total DASS-21 score (t=-2.44, p=0.016).

**Conclusions:**

The results of our study imply that female participants exhibit higher levels of depression, anxiety, and stress during the COVID-19 pandemic. Larger, population-based studies could provide a mroe in-depth answer to the importance of these differences for the general population.

